# Effects of limonene,* n*-decane and* n*-decanol on growth and membrane fatty acid composition of the microalga *Botryococcus braunii*

**DOI:** 10.1186/s13568-018-0718-9

**Published:** 2018-11-28

**Authors:** Eric Concha, Hermann J. Heipieper, Lukas Y. Wick, Gustavo A. Ciudad, Rodrigo Navia

**Affiliations:** 1 Doctoral Program in Science of Natural Resources, University of La Frontera, Av. Francisco Salazar 01145, Temuco, Chile; 2 Department of Environmental Biotechnology, Helmholtz Centre for Environmental Research-UFZ, Permoserstr. 15, 04318 Leipzig, Germany; 3 Department of Environmental Microbiology, Helmholtz Centre for Environmental Research-UFZ, Permoserstr. 15, 04318 Leipzig, Germany; 4 Department of Chemical Engineering, Instituto del Medio Ambiente, Scientific and Technological Bio-resource Nucleus, University of La Frontera, Av. Francisco Salazar 01145, Temuco, Chile; 5 Department of Chemical Engineering, Centre for Biotechnology and Bioengineering (CeBiB), Scientific and Technological Bio-resource Nucleus, University of La Frontera, Av. Francisco Salazar 01145, Temuco, Chile

**Keywords:** *Botryococcus braunii*, Two-phase system, Solvent tolerance, Fatty acid profile, Unsaturation index

## Abstract

*Botryococcus braunii* is a promising microalga for the production of biofuels and other chemicals because of its high content of internal lipids and external hydrocarbons. However, due to the very thick cell wall of *B. braunii*, traditional chemical/physical downstream processing very often is not as effective as expected and requires high amounts of energy. In this cases, the application of two-phase aqueous-organic solvent systems could be an alternative to cultivate microalgae allowing for a simultaneous extraction of the valuable compounds without significant negative effects on cell growth. Two-phase systems have been applied before, however, there are no studies so far on the mechanisms used by microalgae to survive in contact with solvents present as a second-phase. In this study, the effects of the solvents limonene,* n*-decane and* n*-decanol on growth of the microalga *B. braunii* as well as the adaptive cell response in terms of their phospholipid fatty acid contents were analized. A concentration-dependent negative effect of all three solvents on cell growth was observed. Effects were accompanied by changes of the membrane fatty acid composition of the alga as manifested by a decrease of the unsaturation . In addition, an association was found between the solvent hydrophobicity (given as log octanol–water partition coefficient ($\text {P}_{O-W}$) values) and their toxic effects, whereby* n*-decanol and* n*-decane emerged as the most and least toxic solvent respectively. Among the tested solvents, the latter promises to be the most suitable for a two-phase extraction system.

## Introduction

*Botryococcus braunii* is a microalga that can be found in fresh, brackish, and saline water all around the world (Aaronson et al. 1983). This microalga is considered to be a source of lipids and hydrocarbons and can thus possibly serve as a base for renewable fuel production (Ashokkumar and Rengasamy 2012). It is known that lipid productivity in *B. braunii* is higher when it is cultivated under nitrogen-depletion or other stress conditions (Cheng et al. 2013). However, the total amount of lipids available for biotechnological applications depends on the biomass productivity, that is normally reduced under stress conditions. Unlike lipids, hydrocarbon production is proportional to cell growth. Accordingly, more hydrocarbons are obtained when more biomass is produced (Kojima and Zhang 1999).

For extracting biotechnologically valuable products from microorganisms generally two different methods are used: (i) intensive extraction from harvested biomass (Cooney et al. 2009; Kumar et al. 2015) and (ii) continuous extraction in a two-phase aqueous-organic solvent system, during ongoing microbial growth (Kleinegris et al. 2011). This second approach has been used to extract valuable compounds such as carotenoids, lipids, and hydrocarbons from microalgae maintaining, whereby cell growth is, at least partially, maintained (Hejazi and Wijffels 2004; Sim et al. 2001; Zhang et al. 2011a).

Two-phase systems may also be advantageous for the extraction of hydrocarbons from *B. braunii* in a biofuel production context as: (i) most hydrocarbons of *B. braunii* are located outside of the cell wall (approx. 95% according to Largeau et al. (1980)), and are therefore more easily extractable than internal lipids; (ii) two-phase systems potentially allow for both, the ongoing cultivation of cells and the harvest of external hydrocarbons which move from aqueous to solvent phase.

Maintaining microbial growth in a two-phase system depends on the tolerance and adaptive properties of microorganisms to the conditions and solvents applied. Responses of bacteria in contact with solvents have been widely studied (Manefield et al. 2017) and data show that solvent effects on cell membrane include alterations in order, packing, and interaction of lipids–lipids and lipids–proteins, or impairments on membrane functions such as the selective permeability and enzymatic activity (Isken and de Bont 1998; Mattos 2001; Weber and de Bont 1996). Adaptive bacterial responses to counteract solvent effects include alterations of the content of their membrane phospholipid fatty acids, morphological changes, active solvent transport out of cell membrane, and modification of surface charge and hydrophobicity (Guan et al. 2017; Heipieper et al. 2007; Kusumawardhani et al. 2018; Segura et al. 2012).

A convenient proxy for the adaptation of the membrane of eukaryotic cells (including fungi and algae) is the fatty acid unsaturation index (UI) (Heipieper et al. 2000). This index is the average number of double bonds present in every lipid unit in the sample. In this experiment UI is the unsaturation level index of membrane fatty acids. Therefore, a decrease in the UI is related to a decrease in membrane fluidity and an increase in the rigidity of the cell membrane (Weber and de Bont 1996), as a response, for instance, to membrane fluidizing solvents.

Previous studies examining the effect of stress on the fatty acid profile of microalgae include effects of NaCl, irradiation, ${\text {CO}_{2}}$, temperature and heavy metals (Chen et al. 2008; Dawaliby et al. 2016; Kalacheva et al. 2002; McLarnon-Riches et al. 1998; Rao et al. 2007; Sushchik et al. 2003; Vazquez and Arredondo 1991; Yoshimura et al. 2013; Zhila et al. 2011). However, to our knowledge so far no study has addressed the solvent stress on changes of the UI of microalgae membrane fatty acids.

Depending on goals, solvent selection should be a balance among different solvent characteristics (Daugulis 1988). In this study, on the one hand hydrocarbon extraction capabilities and biocompatibility are necessary, but on the other hand a sustainable solvent production and an easy solvent-hydrocarbon separation (low energy cost) are desirable from a renewable fuel production perspective. These last two conditions, are reasons to consider limonene and decane candidates. The former is a non-petroleum derived, renewable solvent (Njoroge et al. 2004), whereas the latter is one of the lowest molecular weight highly biocompatible alkanes (León 2003). Decanol, the alcohol derived from decane, is less hydrophobic and, therefore, more water soluble, what could provide a higher hydrocarbon extraction although a lower biocompatibility. In this study, only biocompatibility will be tested.

Limonene has been used before to extract hydrophobic compounds such as oils and carotenoids from diverse types of matrices with good results (Chemat-Djenni et al. 2010; Mamidipally and Liu 2004; Tanzi et al. 2012; Virot et al. 2008a, b). Oil extraction yields, based on dry weight, have oscillated between 13.1% (Chemat-Djenni et al. 2010) and 48.6% (Virot et al. 2008b), although these values depend on oil content in their respective matrices. Limonene used to extract lipids from the microalga *Chlorella vulgaris* recovered 38.4% of its respective total lipids (Tanzi et al. 2012). Nevertheless, in our knowledge, limonene has never been used as solvent in a two-phase system to extract hydrophobic compounds.

Decane has been used as solvent to extract hydrophobic compounds from two-phase systems with alive microalga. Results have varied in biocompatibility and extraction capacity, oscillating from high (León 2003; León et al. 2001; Zhang et al. 2011b) to low (Hejazi et al. 2002; León et al. 2001) biocompatibility and from acceptable (León 2003; Zhang et al. 2011b) to poor (Mojaat et al. 2008) compound extraction capabilities. These results, however, depend on extraction system conditions and microalga species and should therefore be taken with caution.

In this study, we tested the effects of both mineral solvents,* n*-decane and its derived alcohol* n*-decanol, as well as the effects of the renewable solvent limonene, on the growth and membrane fatty acid profile of the microalga *B. braunii* in a two-phase aqueous-organic solvent system.

## Materials and methods

### Preculture conditions

A 6 L preculture was established to supply biomass in an exponential growth phase for two-phase cultures. The microalga strain used in this experiment was *Botryococcus braunii* race A (UTEX LB572) provided by the Universidad de Antofagasta, Chile. The preculture was carried out in a 10 L glass bottle (Cat.No.11 602 00, Duran Group) using the medium described by Bazaes et al. (2012), but replacing $\text {HPO}_3$ for $\text {NaH}_2\text {PO}_4$. The pH was set at 6.5 using HCl (5 M) and the medium was autoclaved at 121 °C for 21 min. The microalga grew under continuous (24:0 h light:dark cycle) cool fluorescent illumination at ca. $1500\text { lx}$, and 25 ± 1 °C with neither aeration nor ${\text {CO}_{2}}$ source. To prevent microalga precipitation the flasks were shaken twice a day manually.

### Two-phase cultures

The experiment was set up as a two-factor factorial design, with solvent and solvent concentration as factors. When the biomass in precultures reached the exponential growth phase, 48 parts of the culture were taken (100 mL volume) and either limonene,* n*-decane and* n*-decanol were added in the necessary amount to obtain the following solvent concentrations (mM): (1) limonene: 123.3, 12.3, 1.2, 0.6, 0.3; (2)* n*-decane: 513.0, 282.2, 51.3, 28.2, 5.1; (3)* n*-decanol: 157.3, 15.7, 1.6, 0.8, 0.4. Concentrations were determined based upon literature (Frenz et al. 1989b; Liu and Mamidipally 2005; Mojaat et al. 2008; Zhang et al. 2011b) and toxicity assays (OECD 1984). According to the authors reports and pilot studies this range of concentrations produce quick cell death (higher rates among higher concentrations) but also fully functional cells to observe changes in membrane fatty acid composition. Three replicates, placed in 240 mL flasks with rubber caps, were used for each treatment, totalling 48 runs including three control samples (cultures without solvents). After 24 h, two aliquots were taken from every flask, one to measure biomass concentration changes (growth) and the other one to determine membrane fatty acid profile.

All conditions for two-phase cultures were the same as in preculture, including culture media and continuous illumination.

### Cell growth measurement

Cell growth in culture and preculture was determined using a Coulter counter device (isoton II solution as diluent, 100 μm electrode, 1:500 dilution) (Neumann et al. 2005a; Nguyen et al. 2013; Ríos et al. 2012). Samples were taken in the morning, and after that two-phase cultures were shaken manually twice a day (12.00 and 20.00 h), to avoid that solvent droplets in samples modify microalga cell concentrations.

### Solvent concentration in cell membrane

According to Sikkema et al. (1994) there is a direct correlation between the hydrophobicity given as log P values of a solvent and their partitioning in biological membranes. The following empirical relation was estimated: $log(P_{M{-}W})=0.97*log(P_{O{-}W})-0.64$, where $P_{M{-}W}$ and $P_{O{-}W}$ are membrane/water and octanol/water partition coefficients, respectively. This equation allows to calculate solvent concentration in a membrane for a resting-system case, which will be helpful for result interpretation (Neumann et al. 2005b).

### Characterization of membrane fatty acid profile

Membrane fatty acid profile was characterized for the control samples and biomass in contact with solvents 24 h after the first solvent-microalga contact. Membrane lipids were extracted according to Bligh and Dyer (1959) and transformed into fatty acid methyl ester (FAME) as described by Morrison and Smith (1964). FAME identification was performed using a GC-FID Agilent 6890N, equipped with a capillary chromatographic column (CP-Sil 88 capillary column, Chrompack, ID: 0.25 mm, longitude: 50 m, film: 0.2 μm). A proxy for the relevant presence of double bonds in the membrane fatty acid profile was calculated as follows:

1 $${\text{UI}} = {\frac{(\%{\text{C}}16:1 + \%{\text{C}}18:1) + (\%{\text{C}}18:2*2) + (\%{\text{C}}18:3*3)}{100}},$$

where UI is the unsaturation index (Heipieper et al. 2000; Kaszycki et al. 2013).

### Data processing

The experiment was set as a two-factor factorial design, with solvent and solvent concentration as factors. All experiments were carried out in triplicates. The obtained data were analyzed using analysis of variance (ANOVA) to detect significant differences between solvents or solvent concentration effects. The probability of $\alpha$ (type I error) was set at 5%. All data processing and plots were made using the statistical computing software R (version 3.3.3) (R Core Team 2017).

## Results

### Cell growth

**Fig. 1:**
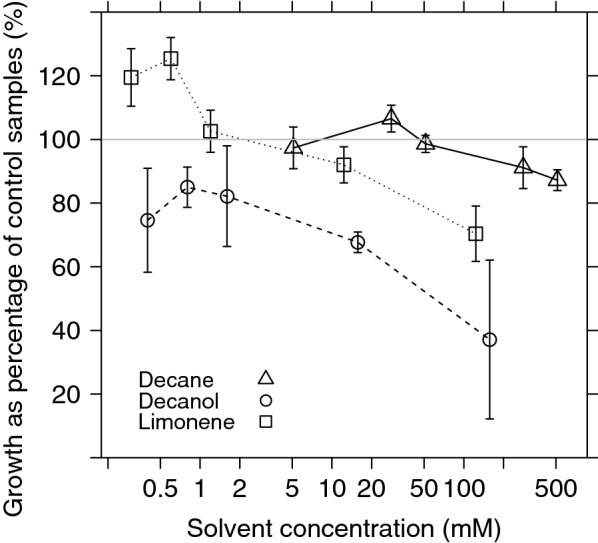
Effect of different concentrations of limonene (square),* n*-decanol (circle) and* n*-decane (triangle) on *Botryococcus braunii* UTEX LB572 growth, after 24 h solvent-biomass contact. Growth is expressed as a percentage of control samples. Every point is the average of three independent samples. Error bars represent standard error of the mean of the same three samples

The effect of three solvents of different log $\text {P}_{O{-}W}$ on *B. braunii* growth was measured (Fig. 1).* n*-decanol (log $\text {P}_{O{-}W}$ = 3.97) was found to be the most toxic solvent tested, resulting in a lower cell concentration compared to* n*-decane and limonene at quasi identical solvent concentrations (p-value < 0.01). In the case of limonene (log $\text {P}_{O{-}W}$ = 4.23), cultures with concentrations lower to 1.2 mM showed higher growth than control samples (p-value = 0.03), i.e., values greater than 100% as illustrated in Fig. 1. Cultures using* n*-decane as second-phase (log $\text {P}_{O{-}W}=5.01$) grew similarly to the control samples up to 51.3 mM of solvent concentration (p-value = 0.89), and then slowly started to decrease to 70% of control sample growth. As expected, the general trend for all the solvents was a lower relative growth when solvent concentration increased and when log $\text {P}_{O{-}W}$ decreased (see Fig. 1).

### Characterization of fatty acid profile from cells in contact with solvents

**Fig. 2:**
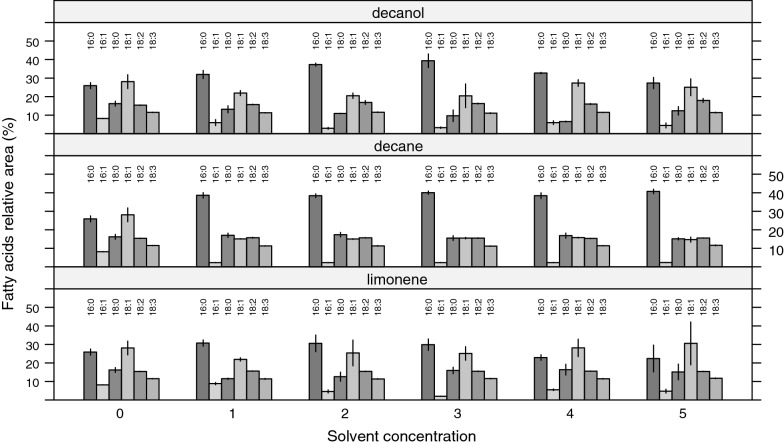
Effect of* n*-decanol,* n*-decane and limonene on membrane fatty acid profile of *Botryococcus braunii* UTEX LB572 after 24 h biomass-solvent contact for 5 different concentrations (1 to 5) and control samples (0). The number 1 correspond to the lowest concentration for every solvent, meanwhile number 5 correspond to the highest one. All data represent the average and standard error of the mean of three independent samples

The fatty acid profile of cells from control samples, revealed that cell membranes of *B. braunii* contain mainly oleic acid (C18:1cis9, 28.0%) and palmitic acid (C16:0, 25.9%). The main effects of solvents on membrane fatty acid profile were observed on C16:0 and C18:1, and to a minor degree on C16:1. C18:2 and C18:3 showed no significant changes (Fig. 2). On the one hand, cells in contact with* n*-decanol and* n*-decane synthesized higher amounts of C16:0 (p-value < 0.01) on average, followed by a decrease in the content of C18:1, especially in decane (p-value < 0.01). On the other hand, limonene presents a monotonic ascending trend for C16:0 and the opposite for C18:1, for increasing solvent concentrations. These changes in fatty acid profile were reflected by different UIs, showing differences in response to both the solvent type and solvent concentration, suggesting predominance of saturated fatty acids and those fatty acids with one double bond.

**Fig. 3:**
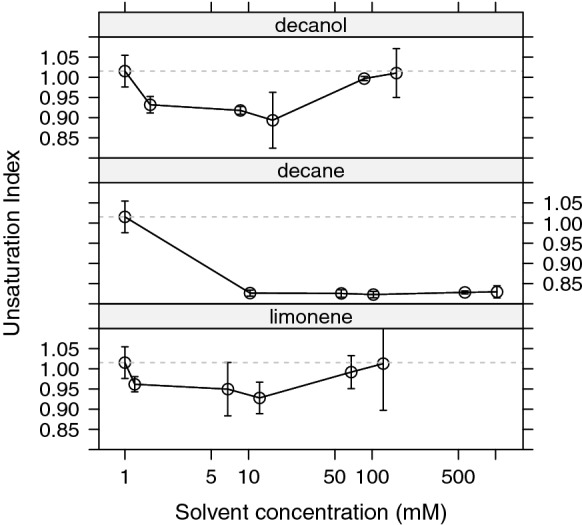
Effect of solvents on membrane fatty acid UI of *B. braunii* UTEX LB572, after 24 h solvent-biomass contact. Slashed horizontal line show control samples UI in every panel. Every point is the average of three independent samples. Bars show standard error of the mean

Addition of* n*-decane resulted in a decreased UI remaining at $UI\approx 0.82$, regardless of the solvent concentration. The presence of limonene and* n*-decanol at the three lowest concentrations levels likewise lowered the UI to the following range: $UI\approx$ 0.90–0.96. For these solvents, two higher concentrations did not result in important changes on the UI compared to the control samples (Fig. 3).

## Discussion

The aim of this study was to test the effects of* n*-decane,* n*-decanol, and limonene on growth and membrane fatty acid composition, in particular the UI of *B. braunii* UTEX LB572 cells. Addition of solvents to *B. braunii* led to differing extents of growth inhibition. At quasi equimolar concentration* n*-decanol was found to be the most toxic solvent followed by limonene and* n*-decane, which showed the least inhibitory effects (Fig. 1). Such data provide valuable information for a better evaluation of the relative physiological status of the cells and associated changes of their fatty acid profile and UIs as will be discussed below.

In 1994, Sikkema et al. (1994) hypothesized that solvent toxicity is primarily governed by the amount of solvent dissolved into the membrane rather than its chemical structure. Thus, the accumulation of molecules in the cell membrane of microorganisms would be the cause of negative effects on bilayer stability, packing of acyl chains and ion leakage problems, resulting in stress, arrest of growth, or even cell death in the extreme case (Weber and de Bont 1996). This hypothesis was supported by results of Heipieper et al. (1995), who, working with different types of solvents on Pseudomonas putida S12, found that the concentration in membrane that produces a 50% loss in growth is similar for all of them: between 60 and 200 mM (solvents used were: methanol, ethanol, 1-butanol, phenol, 1-hexanol, p-cresol, 4-chlorophenol, toluene, 1-octanol, and 2,4-dichlorophenol).

In this study, the membrane solvent concentration was calculated for the maximum water solubility for every solvent, according to the works by Sikkema et al. (1994) and Neumann et al. (2005b). Results in Table 1 show that, in a resting system,* n*-decane reached a maximum membrane concentration (MMC) around 6 mM. A low value compared with the range 60–200 mM. In contrast, MMC for limonene and* n*-decanol were higher than two hundred mM, 294 and 374 mM respectively, suggesting that this is the reason for the low toxicity of* n*-decane and high toxic effects of* n*-decanol on the microalga *B. braunii*. Although this is an approximate estimation of the actual solvent concentration in the cell membrane, it was consistent with results of the growth curves in Fig. 1. These curves show that, on average, solvents with higher log $\text {P}_{O{-}W}$ are more biocompatible. This finding is in line with previous research on *B. braunii* and other microalgae (Frenz et al. 1989a, b; León et al. 2001; Zhang et al. 2011a).

**Table 1:** Physico-chemical properties of solvents used in the two-phase aqueous-organic system

Solvents	Molar mass (g/mol)	Maximum water solubility (mM)	Log $\text {P}_{O{-}W}$^a^	Log $\log {\text{P}}_{{M{{-}}W}}$^b,c^	MMC^d^	$\text {[M]/[M}{_{dec}}]$ ^e^
Decane	142.29	0.000366	5.01^f^	4.22	6	1
Limonene	136.23	0.101299	4.23^g^	3.46	294	$\approx$ 49
*n*-decanol	158.28	0.230000	3.97^h^	3.21	374	$\approx$ 62

${\text{Log}\, \text {P}_{M{-}W}}$ and $\text {[M]/[M}{_{dec}}$] were included as references

^a^ Logarithm of octanol-water partition coefficient

^b^ Logarithm of water-membrane partition coefficient

^c^ Calculated according to Sikkema et al. (1994)

^d^ MMC: maximum membrane concentration of solvent. Calculated according to Neumann et al. (2005b)

^e^ Solvent concentration in membrane (M) divided by* n*-decane concentration in membrane ($\text {M}_{dec}$)

^f^ Data from Mojaat et al. (2008)

^g^ Data from Filipsson et al. (1998)

^h^ Data from Frenz et al. (1989b)

Notably, for some concentrations of limonene and* n*-decane, growth reached values greater than 100%. A possible explanation is that within a certain range of concentration, solvents produced cell membrane instability, which in turn favours mass transfer between cells and culture medium. Consequently, nutrients and oxygen permeate more easily through cell membrane, accelerating growth (León et al. 2001). This high growth associated with limonene and* n*-decane is also in agreement with previous studies on *Aerobacter aerogenes* and *Saccharomyces cerevisae* (Jia et al. 1997; Rols et al. 1990), which reported that oxygen dissolves more easily in organic solvents compared with water, working as improved oxygen-vectors, thus incrementing the oxygen transfer rate and growth in *B. braunii* cultures. Another reason for the high growth could be that solvents are actually working, simultaneously, as carbon sources (de Carvalho and da Fonseca 2004; de Carvalho et al. 2005), which is possible as *B. braunii* has been reported as a mixotrophic microalga (Tanoi et al. 2010; Zhang et al. 2011b).

The adaptive response of *B. braunii* to solvent contact was similar for all solvents tested in our studies. The greatest changes in fatty acid profile were produced by* n*-decane, where C16:0 abundance was remarkably increased while C18:1 decreased. A reduction of C16:1 also occurred (Fig. 2). As a result of these changes, an UI drop from 1.02 (control samples) to around 0.82 for all* n*-decane concentrations was produced (Fig. 3). As Fig. 1 illustrates, cells in contact with* n*-decane seem to have a growth comparable to control samples for all concentrations. These outcomes suggest that* n*-decane, dissolved in culture media and cell membrane, was enough to stimulate cells to produce *de novo* synthesis of saturated and/or less unsaturated lipids to counteract increased fluidity, but not enough to stop cell growth (Figs. 1 and 3).

According to Piper (1995), solvent accumulation in cell membrane produce changes in membrane fatty acids similar to those produced by an increase in temperature, due to both stressors induce an increment in fluidity and loss of selective permeability. *B. braunii* exposed to rising temperatures showed a reduction in its UI (Kalacheva et al. 2002; Sushchik et al. 2003), as was also found in this study. Sushchik et al. (2003) observed that an increment from 30 °C up to 40 °C increased C16:0 from 56.3 up to 73.0% of total fatty acids, while simultaneously C18:2 and C18:3 were reduced from 14.9 to 8.8% and from 19.4 to 10.3%, respectively. In this study, however, there were no significant changes in linoleic (C18:2) or linolenic (C18:3) acid abundance, probably because the increase in membrane rigidity due to the reduction from 3 to 2, or 2 to 1 double bond is not as large as when the change is from 1 to 0 double bond, since the structure of a saturated fatty acid is linear. The underlying logic in a reduction of membrane fatty acid unsaturation is that saturated fatty acids counteract increasing membrane fluidity and permeability, due to rising temperature or solvent contact with cells, as they can be packed more tightly due to their straightness (Sikkema et al. 1995). Other microalgae exposed to a rise in temperature, such as *Nannochloropsis *sp. (Hu and Gao 2016) and *Chlorella vulgaris* (Sushchik et al. 2003) also showed a similar behaviour, reducing unsaturation. Meanwhile a reduction in temperatures, produce the opposite effect, i.e., increased fatty acid unsaturation to maintain membrane fluidity (Chen et al. 2008; McLarnon-Riches et al. 1998; Mikami and Murata 2003; Thompson et al. 1992).

Microalgae can also change fatty acid unsaturation levels to regulate membrane fluidity altered by modifications in environmental or anthropogenic factors such as light, heavy metals, CO_2_ or NaCl. However, the direction of changes are not always clear (Hu and Gao 2016; McLarnon-Riches et al. 1998; Tsuzuki et al. 1990; Zhila et al. 2011) since eukaryotes use others complementary mechanisms to regulate membrane stability, such as production of sterols or synthesis of metabolites to counteract osmotic pressure produced by salts (Rao et al. 2007; Vazquez and Arredondo 1991).

With regard to limonene and* n*-decanol an UI reduction was found (compared to control samples) at the three lower solvent concentrations, meaning cells were still able to perform changes at fatty acids synthesis level, despite of the stress produced by solvents. At the two higher solvent concentrations the UI remained comparable to the control samples for both solvents. Coincidently, higher concentrations of limonene and* n*-decanol produced lower growth rates compared to control samples suggesting that stress reduces synthesis of fatty acids, which is a requisite for a change in the saturated/unsaturated ratio (Segura et al. 2004).

In conclusion, this study confirms for *B. braunii*, what has been known for bacteria: *B. braunii* performs changes in lipid profile and unsaturation of membrane lipids in contact with solvents as a strategy to maintain membrane fluidity, tolerate stress and keep its growth. Additionally, as predicted by log $\text {P}_{O{-}W}$,* n*-decanol was identified as the most aggressive solvent as second-phase; limonene had an intermediate effect, whereas* n*-decane seems to be able to maintain high growth rates even at high concentrations, being the most suitable solvent to extract valuable lipophilic compounds like hydrocarbons in a two-phase culture, under conditions used in this study.

## Abbreviations

FAME: fatty acid methyl ester
lx: lux
mM: milimolar
MMC: maximum membrane concentration
$\text {P}_{O-W}$: partition coefficient octanol-water
$\text {P}_{W-M}$: partition coefficient water-membrane
UI: unsaturation index
